# Hypoxia Regulates CD44 and Its Variant Isoforms through HIF-1α in Triple Negative Breast Cancer

**DOI:** 10.1371/journal.pone.0044078

**Published:** 2012-08-28

**Authors:** Balaji Krishnamachary, Marie-France Penet, Sridhar Nimmagadda, Yelena Mironchik, Venu Raman, Meiyappan Solaiyappan, Gregg L. Semenza, Martin G. Pomper, Zaver M. Bhujwalla

**Affiliations:** 1 JHU ICMIC Program, Division of Cancer Imaging Research, The Russell H Morgan Department of Radiology and Radiological Science, Johns Hopkins University School of Medicine, Baltimore, Maryland, United States of America; 2 Sidney Kimmel Comprehensive Cancer Center, Johns Hopkins University School of Medicine, Baltimore, Maryland, United States of America; 3 Department of Pediatrics, Johns Hopkins University School of Medicine, Baltimore, Maryland, United States of America; Thomas Jefferson University, United States of America

## Abstract

**Background:**

The CD44 transmembrane glycoproteins play multifaceted roles in tumor progression and metastasis. CD44 expression has also been associated with stem-like breast cancer cells. Hypoxia commonly occurs in tumors and is a major cause of radiation and chemo-resistance. Hypoxia is known to inhibit differentiation and facilitates invasion and metastasis. Here we have investigated the effect of hypoxia on CD44 and two of its isoforms in MDA-MB-231 and SUM-149 triple negative human breast cancer cells and MDA-MB-231 tumors using imaging and molecular characterization.

**Methods and Findings:**

The roles of hypoxia and hypoxia inducible factor (HIF) in regulating the expression of CD44 and its variant isoforms (CD44v6, CD44v7/8) were investigated in human breast cancer cells, by quantitative real-time polymerase chain reaction (qRT-PCR) to determine mRNA levels, and fluorescence associated cell sorting (FACS) to determine cell surface expression of CD44, under normoxic and hypoxic conditions. *In vivo* imaging studies with tumor xenografts derived from MDA-MD-231 cells engineered to express tdTomato red fluorescence protein under regulation of hypoxia response elements identified co-localization between hypoxic fluorescent regions and increased concentration of ^125^I-radiolabeled CD44 antibody.

**Conclusions:**

Our data identified HIF-1α as a regulator of CD44 that increased the number of CD44 molecules and the percentage of CD44 positive cells expressing variant exons v6 and v7/8 in breast cancer cells under hypoxic conditions. Data from these cell studies were further supported by *in vivo* observations that hypoxic tumor regions contained cells with a higher concentration of CD44 expression.

## Introduction

Hypoxic tumor microenvironments induce phenotypic changes in cancer cells that make them aggressive [1], [2], refractory to treatment [3], [4], and likely to metastasize [5], [6]. Most of these phenotypic alterations are mediated through a transcription factor belonging to the basic helix-loop-helix PAS superfamily called hypoxia-inducible factor (HIF). HIF is a heterodimer consisting of an oxygen dependent α subunit and a constitutively expressed β subunit. This heterodimer recognizes a 5-bp consensus element (RCGTG) known as hypoxia response element (HRE) on the untranslated regions of over 150 genes, and activates their transcription [2]. Transcriptional activity is achieved following the binding of stabilized HIF-1α protein (or its homolog HIF-2α) to HIF-1β.

CD44 transmembrane glycoproteins are cell adhesion molecules that have been associated with aggressiveness and metastasis [7], [8]. CD44 is also known as cellular adhesion molecule PGP-1, or Hermes antigen [9]. Members of the CD44 family differ in the extracellular domain by the insertion of variable regions close to the transmembrane domain that result in CD44 variant isoforms (CD44v). A common property of all CD44 isoforms is the ability to bind to hyaluronan. In many cancer types, including breast cancer, CD44 and some of its alternate splice variants have been associated with increased invasion, metastasis and with poor prognosis [10], [11]. CD44 has also been identified as a marker of stem-like breast cancer cells [12], [13], although its functional role in this phenotype is not clearly defined.

Recent studies suggest that hypoxia provides a suitable niche for stem cells to maintain their precursor status [14]. Bone marrow-derived endothelial progenitor cells home to hypoxic or injured tissue [15], and the homing of leukaemic stem cells has been found to depend on CD44 [16]. We therefore investigated the relationship between CD44 and hypoxia in triple (estrogen receptor, progesterone receptor and Her2/neu) negative (ER, PR, Her2/neu negative) MDA-MB-231 and SUM-149 human breast cancer cells. We focused on triple negative breast cancer (TNBC) cells in our investigations as TNBC is the most lethal form of breast cancer, and included the well-established inflammatory breast cancer (IBC) cell line SUM-149 [17], [18] in these studies. IBC is a rare but highly aggressive form of breast cancer with poor prognosis and the insights obtained with these studies may identify strategies to improve treatment outcome. MDA-MB-231 breast cancer cells were engineered to stably express tdTomato red fluorescent protein (RFP) under control of HRE, and were characterized for their ability to report on hypoxia *in vivo* as previously described [19]. Multi-modality *in vivo* single photon emission computed tomography (SPECT) and optical imaging were performed on tumors derived from these cells to establish the relationship between hypoxia and the localization of radiolabeled CD44 antibody *in vivo*, which was further validated *ex vivo*. Extensive molecular characterization of cells in culture, including the evaluation of changes in CD44 expression following exposure to hypoxia or to the hypoxia mimetic cobalt chloride (CoCl_2_) in cells transduced with HIF-1 or -2α short hairpin RNA (shRNA), was performed.

The data collectively identified, for the first time, HIF-1α as a regulator of CD44 expression in breast cancer cells and tumors. Two variant isoforms, CD44v6 and CD44v8, were found to be strongly regulated by hypoxia. Increased expression of CD44 may be an additional mechanism by which hypoxia mediates a more aggressive phenotype. These data also suggest that cells in hypoxic environments have increased expression of a breast cancer stem cell marker, and that hypoxia may partly contribute towards engendering a stem-like phenotype in tumors.

## Materials and Methods

### Cell Culture and Hypoxia Treatment

SUM-149 cells (Asterand, Inc., Detroit, MI) were maintained in Ham’s F12 medium with 5% calf serum, insulin (5 µg/ml), and hydrocortisone (1 µg/ml). MDA-MB-231 wild-type breast cancer cells (ATCC, Manassas, VA) and genetically engineered sublines transduced by lentivirus to stably express either HIF-1α-shRNA or HIF-2α-shRNA were maintained in RPMI 1640 medium (Mediatech, Manassas, VA) supplemented with 10% fetal bovine serum (Sigma, St. Louis, MO), 1% penicillin and streptomycin (Invitrogen, San Diego, CA), and maintained at 37°C in a CO_2_ incubator. MDA-MB-231 cells expressing tdTomato RFP under the control of HRE were cultured in RPMI 1640 medium with 500 µg/ml of G418 (Invitrogen). Hypoxic treatment of cells was performed either by placing the plates in a modular incubator chamber (Billups-Rothenberg, Del Mar, CA), flushed at 2 p.s.i. for 3 minutes with a gas mixture of 0.2% O_2_, 5% CO_2_, and N_2_ for the balance, or by treating cells with 200 µM of CoCl_2_ to chemically mimic hypoxia. mRNA expression levels of vascular endothelial growth factor (VEGF), a well-established downstream target gene that is activated in response to hypoxia through the HIF pathway, were characterized to confirm stabilization of HIF following induction of hypoxia or treatment with CoCl_2_.

### Lentiviral Vector, shRNA and HIF-1α-dominant Negative Construct

The lentiviral construct pRRL-PGK-GFP, production of viral supernatant, and the transduction method used have been previously described [20]. The sequence for HIF-1α-shRNA was obtained from an earlier report [21]. HIF-2α-shRNA in the pLKO vector was obtained from Thermo Scientific (Open Biosystems, Huntsville, AL), and cloned into a pRRL vector. Viral supernatant obtained from the parental vector pRRL-pGK-GFP that lacked any shRNA was used to generate the empty vector (EV) control cells. All constructs were sequence-verified.

### Immunoblot Assay

Whole cell extracts were prepared in radioimmune precipitation (RIPA) buffer, fractionated by SDS-PAGE, transferred to a nitrocellulose membrane, and subjected to immunoblot assays using a mouse monoclonal primary antibody (Novus Biologicals, Littleton, CO) for HIF-1α (NB-100-105, dilution 1∶1000) and for HIF-1β (NB-100-124, dilution 1∶1000) that was used as a loading control, and a rabbit polyclonal antibody for HIF-2α (NB-100-122, dilution 1∶500). Horseradish peroxidase-conjugated secondary antibody against either mouse or rabbit IgG was used at 1∶2500 dilution. The signal was visualized using ECL Plus reagents (Thermo Scientific, Rockford, IL).

### RNA Isolation, cDNA Synthesis, and Quantitative Reverse Transcription-PCR

Total RNA from MDA-MB-231 and SUM-149 cells maintained for 24 h under normoxia, or hypoxia, or treated for 24 h with medium containing 200 µM CoCl_2_, were isolated and the corresponding cDNA was synthesized using qScript (Quanta Bioscience, Gaithersburg, MD) [20]. Quantitative real-time PCR (q-RT-PCR) was performed using IQ SYBR Green Supermix and gene-specific primers in the iCycler RT-PCR detection system (Bio-Rad, Hercules, CA) with 2 µl of diluted cDNA samples (1∶10) used as a template. All primers were designed using Beacon Designer software 5.1 (Premier Biosoft International, Palo Alto, CA). The expression of target RNA relative to the housekeeping gene, hypoxanthine phosphoribosyltransferase 1 (HPRT1), was calculated based on the threshold cycle (Ct) as R = 2^−Δ(ΔCt)^, where ΔCt = Ct_target_ – Ct _HPRT1_ and Δ (ΔCt) = ΔCt_0.2%_ −ΔCt_20%_.

### Fluorescence Associated Cell Sorting (FACS) Analysis of CD44v6, and CD44v7/8

Wild type MDA-MB-231 or genetically engineered MDA-MB-231 cells expressing either HIF-1α- or HIF-2α-shRNA-green fluorescent protein (GFP) were seeded at a density of 1×10^6^ in a 100 mm tissue culture dish. Twenty-four hours later, cells were treated with 200 µM of CoCl_2_ for 48 h. The following monoclonal antibodies were used: anti-CD44v6-PE (phycoerythrin) (R&D systems, Minneapolis, MN), and anti-CD44v7/8-FITC (fluorescein isothiocyanate) (BenderMedSystems, San Diego, CA). Briefly, cells were washed and harvested using PBS EDTA 5 mM buffer. Approximately 0.5×10^6^ cells were resuspended in PBA buffer (PBS containing 0.5% bovine serum albumin and 0.02% sodium azide). Cells were incubated with different antibodies in the dark at 4°C for 45 minutes and washed several times in PBA. Flow analyses were performed using a FACScalibur system (Becton Dickinson Immunocytometry Systems, San Jose, CA). No-antibody controls were analyzed to delineate the unstained and autofluorescing population. Single-antibody controls were used to compensate the sample reading and to designate the quadrants. The percentage of positive events and the mean intensity of fluorescence values were measured using Cell Quest software (Becton Dickinson Immunocytometry Systems, San Jose, CA).

**Figure 1:**
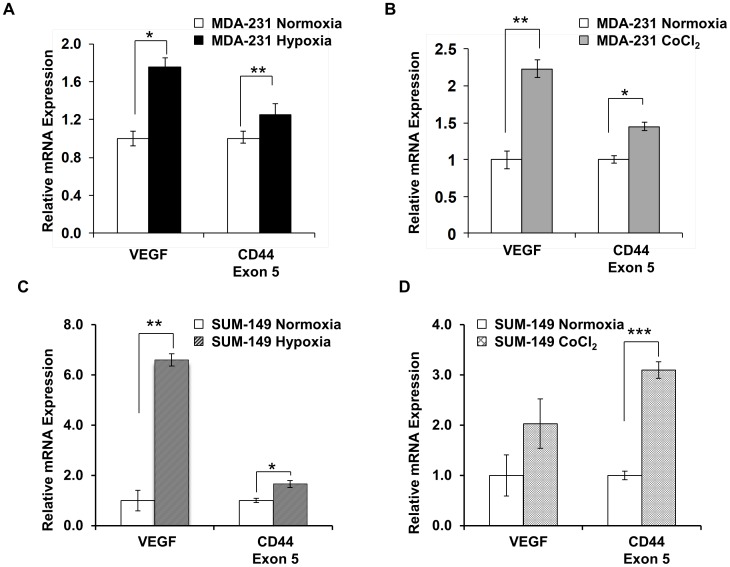
VEGF and CD44 mRNA expression in human breast cancer cell lines. q-RT-PCR analysis of VEGF and CD44 mRNA expression levels in MDA-MB-231 wild type cells under normoxia (open box), in response to 24 h of hypoxia (black box) (A), and in response to 24 h treatment with 200 µM CoCl_2_ (dark-gray box) (B). mRNA expression of VEGF and CD44 in SUM-149 wild type cells under normoxia (open box) and in response to 24 h of hypoxia (hatched box)(C), and in response to 24 h treatment with 200 µM CoCl_2_ (cross-hatched box)(D). Values represent Mean ± SEM obtained from three independent experiments. *P<0.05, **P<0.005.

### Cloning and Establishment of MDA-MB-231 HRE-RFP Cells and Tumors

Cloning of HRE-driven GFP into cancer cells, and the ability of GFP to report on hypoxia in tumors *in vivo*, has been previously reported [19]. Here the GFP gene in the HRE-GFP vector was replaced with the tdTomato RFP gene and sequence verified. MDA-MB-231 cells were transfected with plasmid encoding HRE-RFP using Lipofectamine 2000 (Invitrogen, Carlsbad, CA). Forty-eight hours post-transfection, cells were treated with 500 µg/ml of G418 (Invitrogen, Carlsbad, CA). Various clones were obtained and tested for RFP expression in response to 200 µM CoCl_2_. Pooled clones with brightest RFP expression were sorted by FACS for subsequent experiments. Approximately 2×10^6^ MDA-MB-231-HRE-RFP cells in 50 µl of Hanks balanced salt solution were inoculated in the upper-right thoracic mammary fat pad of age-matched female severe combined immunodeficient (SCID) mice.

### In vivo Detection of CD44, Iodination of CD44 Antibody, SPECT Imaging, and Optical Imaging of HRE-driven tdTomato RFP Expression

#### Antibody labeling

Antibodies were labeled following published protocols [22] with slight modifications. Briefly, 1–2 mCi of [^125^I]NaI were incubated with 30 µg of purified mouse monoclonal anti-human CD44 antibody (A3D8, Abcam Inc., Cambridge, MA) in 50–100 µl PBS in an iodogen-coated reaction vial. Reaction times varied from 10 to 20 min. Radiolabeled antibodies were purified from other low molecular weight impurities using size exclusion chromatography on a Sephadex G-25 desalting column (Amersham Biosciences, Piscataway, NJ) preconditioned with PBS at pH 7.4. Typical radiochemical yields ranged from 40 to 60%. Protein purity, as determined by instant thin layer chromatography, was greater than 95%. Antibodies with a specific radioactivity of 50 µCi/µg per 0.2 ml of saline were administered intravenously.

#### SPECT/CT imaging

A SPECT/CT scanner (Gamma Medica X-SPECT, Northridge, CA) was used for image acquisition. Images were acquired at 48 h post-intravenous injection of 1 mCi of radiolabeled antibody. The SPECT projection data were acquired using two low energy high-resolution parallel-hole collimators with a radius-of-rotation of 3 cm. The tomographic data were acquired in 64 projections over 360 degrees at 40 s/projection. In case of *ex vivo* tumor slices, 80 s/projection was used. Following tomography, CT images were acquired in 512 projections to allow co-registration. Data were reconstructed using the ordered subsets-expectation maximization (OS-EM) algorithm and analyzed using AMIDE software.

#### Optical imaging

Fluorescence imaging of tumors was performed *in vivo* with a Xenogen IVIS Spectrum system (Caliper Life Sciences, Hopkinton, MA). Endpoint fluorescence imaging of fresh 2-mm thick tumor slices prepared with a tissue slicer was performed with the Xenogen system or using a 1× objective on a fluorescence microscope (Nikon Ltd., Melville, NY).

All images were processed using ImageJ v1.34s (freeware for windows developed at the NIH).

**Figure 2:**
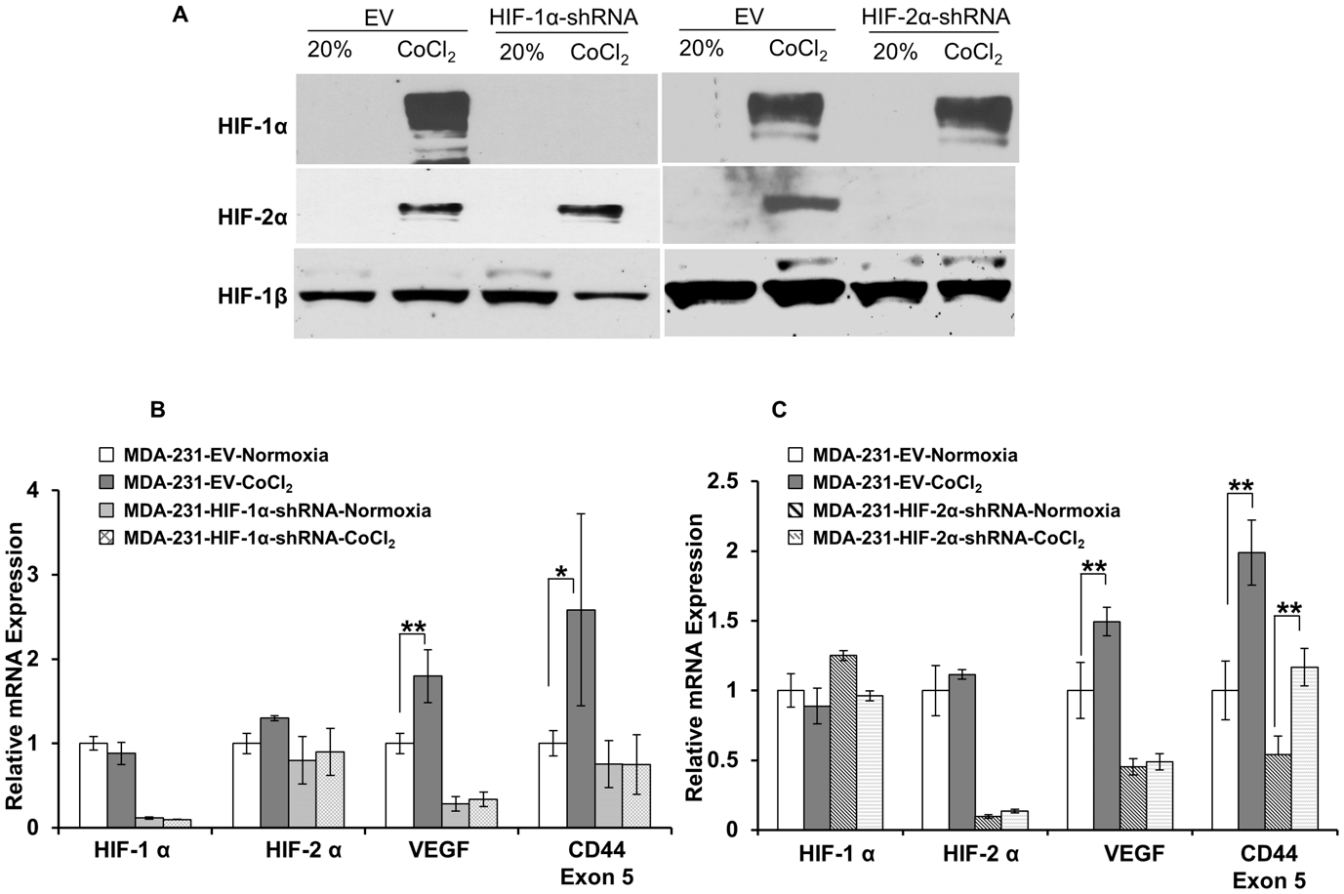
Expression of CD44 mRNA in genetically engineered MDA-MB-231 cells. (A) Immunoblot analysis of HIF-1α or HIF-2α expression in whole cell extracts from MDA-MB-231 cells stably expressing EV, HIF-1α-shRNA or HIF-2α-shRNA under normoxia or in response to 4 h treatment with 200 µM CoCl_2_. HIF-1β expression was used as a loading control. (B) mRNA expression of HIF-1α, HIF-2α, VEGF, and CD44 in MDA-MB-231 EV cells under normoxia (open box) or following 24 h of 200 µM CoCl_2_ (dark-gray box) and in MDA-MB-231 cells stably expressing HIF-1α-shRNA under normoxia (light-gray box) or following 24 h of 200 µM CoCl_2_ (cross-hatched box). (C) mRNA expression of HIF-1α, HIF-2α, VEGF, and CD44 in MDA-MB-231 EV control cells under normoxia (open box) or following 24 h of 200 µM CoCl_2_ (dark-gray box) and in MDA-MB-231 cells stably expressing HIF-2α-shRNA under normoxia (hatched box) or following 24 h of 200 µM CoCl_2_ (wavy-hatched box). Values represent Mean ± SEM of three experiments. *P<0.05, **P<0.005.

### Animal Ethics

All surgical procedures, animal handling and radiolabeling were performed in accordance with protocols approved by the Johns Hopkins University Institutional Animal Care and Use Committee, and conformed to the Guide for the Care and Use of Laboratory Animals published by the NIH.

### Statistical Analysis

Data presented are Mean ± standard error mean (SEM) of a minimum of three experiments. Statistical analysis was performed with Microsoft Excel Software® (Redmond, WA) using a two-sided t-test, assuming unequal variance. Values of *P≤0.05* were considered significant, unless otherwise stated.

**Figure 3:**
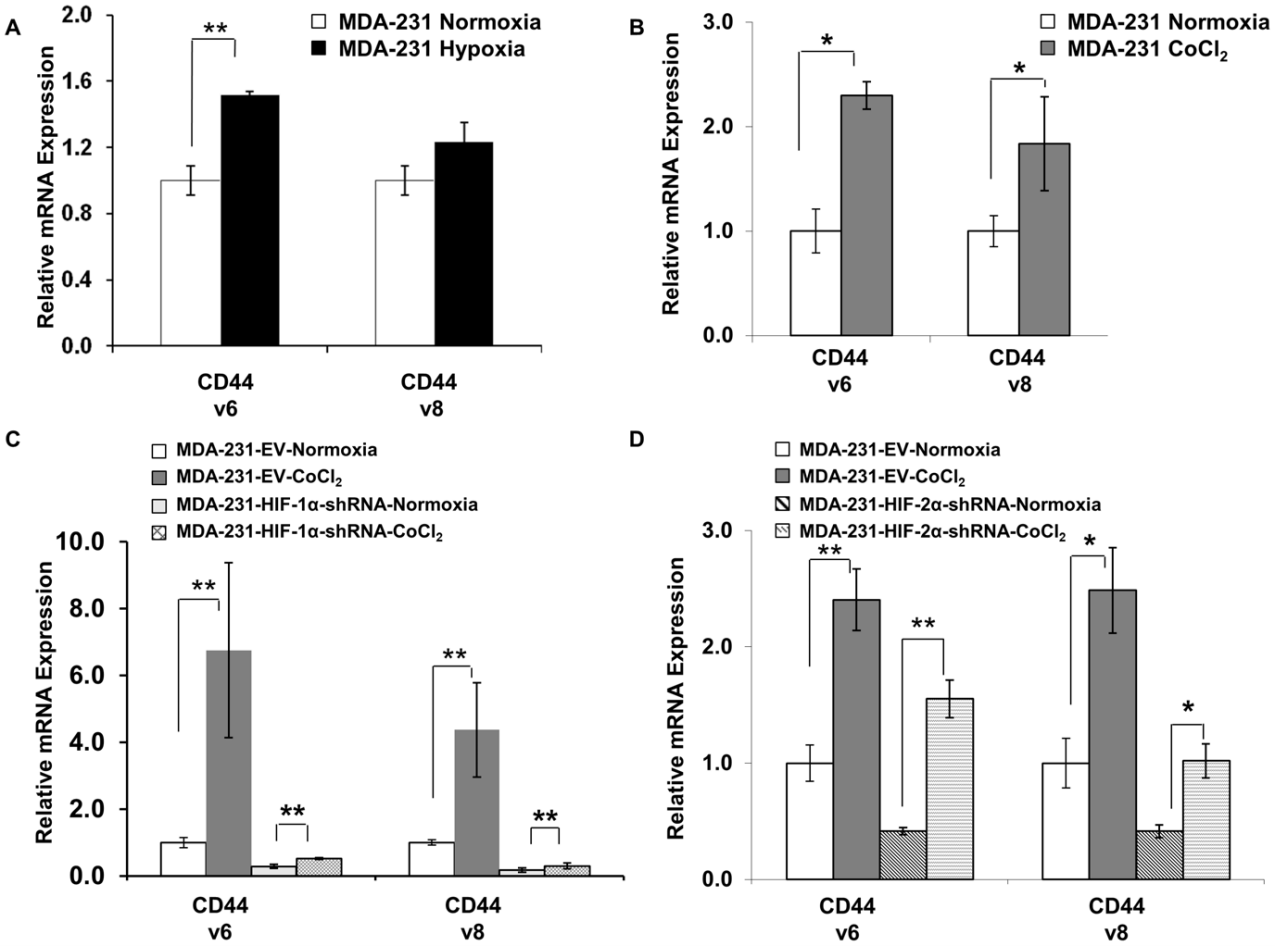
Quantitative real-time PCR analysis of CD44 variant isoforms. mRNA expression levels in MDA-MB-231 wild type cells (A) under normoxia (open box) and in response to 24 h of hypoxia (black box), and (B) under normoxia (open box) and in response to 24 h treatment with 200 µM CoCl_2_ (dark-gray box). (C) mRNA expression of CD44v6 and CD44v8 in MDA-MB-231 EV cells under normoxia (open box) or following 24 h of 200 µM CoCl_2_ (dark-gray box) and MDA-MB-231 cells stably expressing HIF-1α-shRNA under normoxia (light-gray box) or following 24 h of 200 µM CoCl_2_ (cross-hatched box). (D) mRNA expression of CD44v6 and CD44v8 in MDA-MB-231 EV cells under normoxia (open box) or following 24 h of 200 µM CoCl_2_ (dark-gray box) and in MDA-MB-231 cells stably expressing HIF-2α-shRNA under normoxia (hatched box) or following 24 h of 200 µM CoCl_2_ (wavy-hatched box). Values represent Mean ± SEM obtained from three independent experiments. ^#^P<0.07, *P<0.05, **P<0.005.

## Results

### Hypoxia and HIF-1 Regulate CD44 Expression

To determine if hypoxia modulates expression of CD44, qRT-PCR was performed using RNA from MDA-MB-231 and SUM-149 breast cancer cells that were maintained under normoxia (21% oxygen) or hypoxia (1% oxygen), or treated with 200 µM CoCl_2_. Both MDA-MB-231 and SUM-149 cells showed an increase in relative mRNA levels of CD44 and VEGF, a well-established downstream target gene that is activated in response to hypoxia (Figures 1A and C). A significant increase of CD44 and VEGF was also observed in response to the hypoxia mimetic CoCl_2_ (Figures 1B and D).

To characterize the regulation of the CD44 gene, we established two genetically modified sublines of MDA-MB-231 cells with HIF-1α or HIF-2α silenced. These knockdown cell lines were tested for efficient silencing of HIF-1α or HIF-2α protein (Figure 2A) and mRNA (Figures 2B and C) levels in response to treatment with CoCl_2_, using EV cells as controls. Unlike EV cells, HIF-1α or HIF-2α protein expression was not detected in sublines that stably expressed HIF-1α-shRNA or HIF-2α-shRNA, in response to CoCl_2_ (Figure 2A). Expression levels of mRNA of HIF-1α or HIF-2α were significantly downregulated in the corresponding HIF-1α-shRNA (Figure 2B) or HIF-2α-shRNA (Figure 2C) expressing sublines. CD44 mRNA was significantly lower in cells expressing HIF-1α shRNA, and did not increase upon exposure to CoCl*_2_* (Figure 2B). In contrast, a significant increase of CD44 mRNA was observed with CoCl_2_ exposure in HIF-2α-shRNA expressing cells (Figure 2C), identifying HIF-1α, and not HIF-2α, as an important regulator of CD44 under hypoxic conditions.

**Figure 4:**
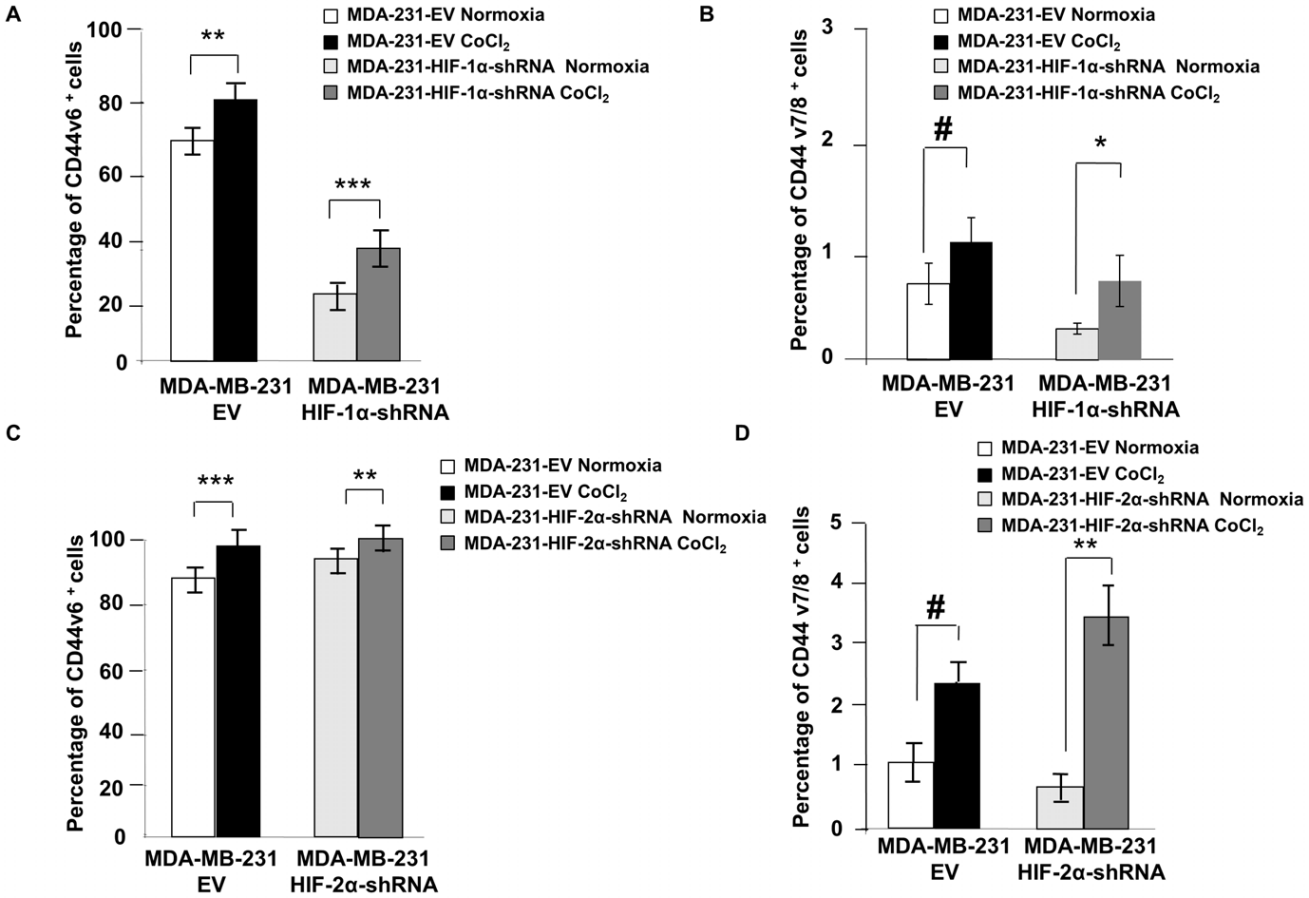
Cell surface expression of CD44 variant isoforms. (A) FACS analysis showing percentage of CD44v6 positive cells in normoxic MDA-MB-231 EV cells (open box), and MDA-MB-231 cells stably expressing HIF-1α-shRNA (light-gray box), or after 48 h of 200 µM CoCl_2_ treatment, in MDA-MB-231 EV (black box) and MDA-MB-231 cells stably expressing HIF-1α-shRNA (dark-gray box). (B) FACS analysis showing percentage of CD44v7/8 positive cells in MDA-MB-231 EV cells (open box), and in MDA-MB-231 cells stably expressing HIF-1α-shRNA under normoxia (light-gray box) or following 48 h treatment with 200 µM CoCl_2_ in MDA-MB-231 EV cells (black box) and in MDA-MB-231 cells stably expressing HIF-1α-shRNA (dark-gray box). (C) FACS analysis showing percentage of CD44v6 positive cells in MDA-MB-231 EV cells (open box), and MDA-MB-231 cells stably expressing HIF-2α-shRNA under normoxia (light-gray box) or following 48 h of 200 µM CoCl_2_ in MDA-MB-231 EV cells (black box) and MDA-MB-231 cells stably expressing HIF-2α-shRNA (dark-gray box). (D) FACS analysis showing percentage of CD44v7/8 positive cells in MDA-MB-231 EV cells (open box), and MDA-MB-231 cells stably expressing HIF-2α-shRNA under normoxia (light-gray box) or following 48 h of 200 µM CoCl_2_ in MDA-MB-231- EV (black box) and MDA-MB-231-HIF-2α-shRNA (dark-gray box). Values represent Mean ± SEM of three experiments. *^#^P<0.07,* *P<0.05, **P<0.01, ***P<0.005.

### Hypoxia and HIF-1α Regulate Alternate Splice Variants of CD44

To determine the regulation of the variant isoforms of CD44 under hypoxia in MDA-MB-231 cells, q-RT-PCR screening using exon-specific primers was performed for variant isoforms 1, 5, 6, 8, 9 and 10. The mRNA expression of variant isoforms 6 (CD44v6) and 8 (CD44v8) significantly increased in response to hypoxia (Figure 3A) or following treatment with CoCl_2_ (Figure 3B) in MDA-MB-231 cells. Similarly, SUM-149 cells treated with CoCl_2_ showed a significant increase of CD44v6 and CD44v8 mRNA expression levels (1 *vs* 3.7-fold for CD44v6, n = 3, *P*<0.0018; 1 *vs* 2.3-fold for CD44v6, n = 3, *P*<0.0006, data not shown). Expression levels of mRNA were significantly downregulated in MDA-MB-231 cells with HIF-1α-shRNA. A small increase in CD44v6 and CD44v8 mRNA was observed in HIF-1α silenced cells following exposure to CoCl_2_ compared to HIF-1α silenced cells under normoxia, although the overall levels even with CoCl_2_ treatment were ten-fold lower compared to EV cells treated with CoCl_2_ (Figure 3C). Downregulation of CD44v6 and CD44v8 mRNA levels was not as evident in HIF-2α-shRNA expressing cells, and the response to CoCl_2_ was more robust compared to HIF-1α-shRNA expressing cells (Figure 3D).

These changes in mRNA levels were reflected in the FACS analyses of CD44v6 and CD44v8 expression. Since the antibody that was used to determine the percentage of CD44v8 positive cells also recognized exon 7, the data for CD44v8 is presented as CD44v7/8. In response to hypoxia, the percentage of CD44v6 positive cells increased significantly in MDA-MB-231 EV cells compared to those maintained under normoxic condition (Figures 4A and B). In cells expressing HIF-1α-shRNA, the percentage of CD44v6 and CD44v7/8 cells decreased significantly compared to EV cells under both normoxic and hypoxic conditions (Figures 4A and B). An increase in the percent of CD44v6 and CD44v7/8 cells was observed with exposure to CoCl_2_ in HIF-1α silenced cells but the overall levels were again significantly lower, especially for CD44v6 expressing cells (Figures 4A and B). In contrast, expression of HIF-2α-shRNA did not decrease the percentage of CD44v6 or CD44v7/8 positive cells (Figures 4C and D), and a robust response to CoCl_2_ was observed in terms of an increase of the percentage of cells expressing CD44v6 and CD44v7/8 in response to CoCl_2_ (Figures 4C and D). These data again identify HIF-1α and not HIF-2α as the primary regulator of CD44v6 and CD44v8 expression.

### CD44 Expression is Higher in Hypoxic Tumor Regions

**Figure 5:**
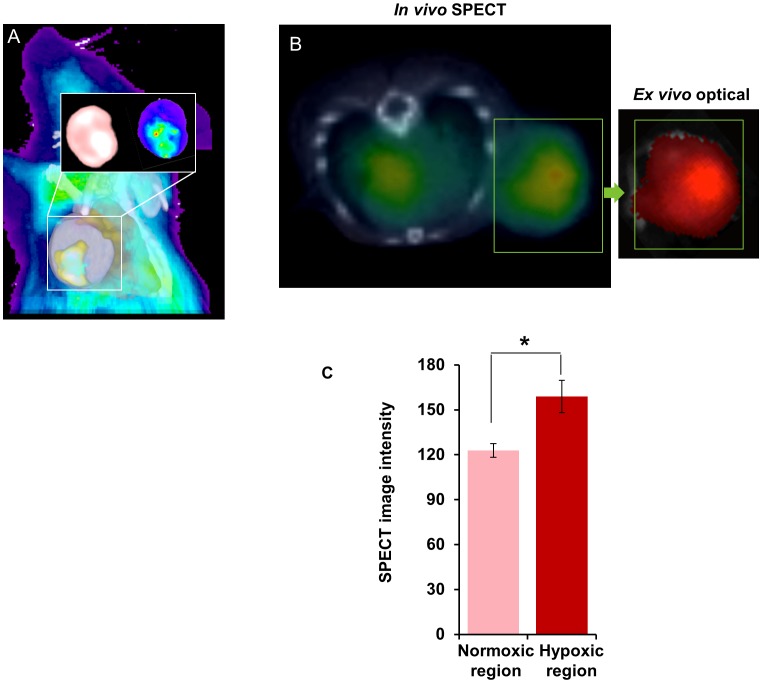
In vivo SPECT and optical imaging to detect CD44 expression. (A) Co-registration of RFP expression from optical images (blue), and SPECT images (yellow) showing the overlap of a hypoxic region of high fluorescence with high CD44 antibody localization. Inset shows *ex vivo* SPECT (left) and optical (right) images of a fresh 2-mm slice from the tumor. (B) *In vivo* SPECT imaging of a SCID mouse bearing MDA-MB-231-HRE-tdTomato RFP tumor was performed in 64 projections at 30 sec/projection. Following SPECT acquisition, CT images were acquired in 512 projections to allow coregistration. Volume rendered images were created using Amira image processing software. A representative trans-axial slice of decay corrected, volume rendered SPECT/CT images at 48 h demonstrate specific accumulation of radioactivity in the tumor as well as some radioactivity in the liver. The optical image of the excised tumor shows a hypoxic region with intense red fluorescence. (C) Quantification of average intensity from SPECT data obtained from normoxic and hypoxic regions in *ex vivo* tissue slices demonstrates a statistically significant increase (*P<0.05, n = 3) in image intensity in hypoxic regions. Values represent Mean ± SEM from 10 slices obtained from three tumors.

*In vivo* SPECT and optical imaging of MDA-MB-231 tumors that express tdTomato RFP under hypoxic conditions identified increased binding of CD44 antibody in hypoxic tumor regions *in vivo*, as shown in Figures 5A and B. This co-localization was confirmed by relating the SPECT images acquired *in vivo* and *ex vivo* to optical images acquired from *ex vivo*, as shown in Figure 5B and summarized in Figure 5C. Image analyses of 10 slices obtained from three tumors revealed significantly higher CD44 antibody localization in hypoxic tumor regions.

## Discussion

TNBCs are a difficult to treat subgroup of cancers that have been observed to express high levels of CD44 [23]. We chose two TNBC cell lines that expressed different levels of CD44 to determine the reproducibility of the increase of CD44 observed with hypoxia. Since approximately 5% of SUM-149 cells express CD44 [24], and approximately 87% of MDA-MB-231 express CD44 [25], these two cell lines represent a wide range of CD44 expression. Both SUM-149 and MDA-MB-231 cells showed an increase in mRNA expression of CD44 and its variant isoforms in response to hypoxia or treatment with CoCl_2_. Since recent reports suggest that cells with high CD44 expression exhibit increased metastatic potential and are resistant to chemotherapy [24], we focused on genetically engineering MDA-MB-231 cells to further understand the mechanisms underlying the response of CD44 to hypoxia. At least two isoforms of CD44, CD44v6 and CD44v8, were found to be strongly regulated by hypoxia and HIF-1α in MDA-MB-231 cells. Additionally, *in vivo* and *ex vivo* imaging identified significantly higher binding of CD44 antibody to cells in hypoxic regions of MDA-MB-231 tumors.

The molecular imaging-detected higher CD44 expression in hypoxic tumor regions *in vivo* and *ex vivo* could be due to hypoxia driving up CD44 expression, or due to hypoxic environments attracting CD44 expressing cells. The latter is plausible since hypoxia drives up CXCR4 and its ligand SDF-1 that mediate cell homing in which CD44 plays a major role as a cell adhesion molecule [26], [27]. However, since nearly 80% or more of MDA-MB-231 cells express CD44 in culture [24], most cancer cells in the tumor will have CD44 expression as was apparent from the SPECT imaging data. In addition, cycling hypoxia has been shown to increase the CD44^+^/CD24^−^ population in a metastatic breast cancer cell line [28]. Therefore, it seemed more likely that CD44 expression was regulated by hypoxia, which was supported by the data obtained here. Although the increase in CD44 expression in hypoxic tumor environments of MDA-MB-231 tumors was significantly higher, it occurred within the backdrop of an already high CD44 expression. It is possible that breast tumors with inherently low CD44 expression may show a more dramatic increase in CD44 expression in hypoxic regions compared to normoxic regions.

Through HIF-1/2α, hypoxia transcriptionally controls a host of factors that contribute to increased resistance to radiation and chemotherapy, and to the emergence of a more aggressive phenotype [3], [11], [29]–[31]. Here we identified CD44, CD44v6 and CD44v8 upregulation by hypoxia as additional factors that may contribute to this phenotype. Since CD44 is also a breast cancer stem cell marker, our data suggest that, *in vivo,* hypoxic tumor regions may contain cell populations enriched with this marker.

Aberrant expression and presence of multiple high molecular weight isoforms of CD44 that are generated by alternate splicing are frequently encountered in breast, colon and gastric cancers [10], [11], [32], [33]. Increased expression of CD44s, the most common isoform of CD44, has been observed in invasive ductal carcinoma, but has been found to have a positive effect on patient survival [8], [34], [35]. However, increased expression of CD44v7/8 has been associated with significantly poorer disease-free survival [33]. CD44v6 [11], [36] and CD44v8 [33] have been directly implicated in tumor progression and poor patient outcome. In our study we observed increased cell surface expression of both CD44v6 and CD44v7/8 and an increase in number of cells that express these markers in response to CoCl_2_. Further studies are necessary to determine the mechanism of alternate splicing in response to hypoxia and the preferential activation of splice variants of CD44.

The functional effects of the upregulation of CD44 and its variant isoforms under hypoxic conditions, especially within the context of hyaluronan levels, should be considered, since cell signaling events that promote anchorage-independent tumor cell growth, survival, migration and metastasis occur through the binding of hyaluronan with CD44 [37], [38]. Upregulation of both CD44 and hyaluronan under hypoxic conditions would most likely amplify these signaling events. Previous studies have reported an increase of hyaluronan and hyaluronidase under hypoxia in T47D breast cancer cells [39]. An increase of CD44 protein was observed in Caki-1 renal cancer cells following hypoxia, which was attributed to the Rho kinase-mediated activation of ERM (Ezrin, Radixin, Moesin) proteins during hypoxia, since ERM proteins are associated with the cytoplasmic domain of CD44 [40].

Here we have established the role of HIF-1α in regulating CD44 in MDA-MB-231 and SUM-149 breast cancer cells, and two of its variant isoforms in MDA-MB-231 breast cancer cells. Future studies should determine the existence of this regulation of CD44 in multiple cell lines across different genetic backgrounds.
